# Trends and Development in Enteral Nutrition Application for Ventilator Associated Pneumonia: A Scientometric Research Study (1996–2018)

**DOI:** 10.3389/fphar.2019.00246

**Published:** 2019-03-20

**Authors:** Shengqi Chen, Ruixue Bie, Yunfeng Lai, Honghao Shi, Carolina Oi Lam Ung, Hao Hu

**Affiliations:** State Key Laboratory of Quality Research in Chinese Medicine, Institute of Chinese Medical Sciences, University of Macau, Macau, China

**Keywords:** ventilator associated pneumonia, enteral nutrition, scientometric, CiteSpace, risk factor, clinical guideline, intensive care unit

## Abstract

**Background:** This study aimed to explore the intellectual landscape of the studies investigating the clinical application of enteral nutrition (EN) in patients with ventilator associated pneumonia (VAP), and to identify thematic development trends and research frontiers in this area.

**Methods:** Scientometric research was conducted by analyzing bibliographic records retrieved from the Web of Science Core Collection Database dated between 1996 and 2018. Reference co-citation analysis, key words co-occurrence analysis and cooperation network analysis were performed using CiteSpace software.

**Results:** A total of 124 valid records were included in the final dataset. It was found that early studies were mainly focused on the feeding pathways of EN among VAP patients. The risks associated with EN intervention in VAP patients, including gastric nutrition intolerance and aspiration pneumonia, were extensively investigated and reported. While aspiration pneumonia has remained a long-term active research area in the field of EN interventions for VAP patients, with recent research focused more on interventions aiming to improve EN support and to optimize the use of EN for VAP patients. It seems that clinical guidelines on EN interventions for VAP patients need to be established.

**Conclusion:** The advantages of EN for VAP patients have been recognized but still require further investigation on standardizing the use. Strategic cooperation among hospital physicians, university researchers and industrial product developers is required to establish clinical guidelines and to continue developing innovative EN products to tackle VAP.

## Introduction

Ventilator associated pneumonia (VAP) is a prevalent and serious disease caused by hospital infection. It usually happens to patients who have been mechanically ventilated for more than 48 h in an intensive care unit (ICU) (Torres et al., 2017). Epidemiology studies estimated that VAP accounted for 9–27% of all mechanically ventilated patients, and the VAP incidence rate ranged between 1.2 and 8.5 cases per 1,000 ventilator days (Kalanuria et al., 2014). The mortality rate of VAP was between 20 and 50% reportedly, and might increase up to 70% (Zubair et al., 2018). Previous studies also showed that patients with VAP had longer durations of mechanical ventilation, ICU stay and hospitalization, compared to those without VAP. Moreover, the costs of healthcare related to VAP treatment have increased significantly over the past few years. In the United States, the average hospitalization cost per VAP patient has increased by 40,000 USD. In Turkey, the average cost of ICU patients with VAP has increased fourfold (Torres et al., 2017). How to effectively prevent and treat VAP remains a medical challenge.

Enteral nutrition (EN) is used in critically ill patients requiring intensive care to provide nutrition support and to reduce the incidence of nutrition-related complications such as infections (Kreymann et al., 2006). It is believed that EN used in ventilator patients can help to reduce the incidence of VAP (Heyland et al., 2003). Furthermore, malnourished patients who have weakened respiratory muscles are at increased risks of contracting VAP as it is difficult to take off ventilator. Therefore, nutritional support treatment, including parenteral nutrition or EN, is important for these patients as it can help to correct hypoproteinemia, and maintain the water, electrolyte and acid-base balance (McClave et al., 2009). In particular, considering its benefits to the digestive system and long-term advantages for patient recovery, EN is now often recommended for these patients (Martindale et al., 2009). In the past two decades, there has been increasing research surrounding the use of EN to reduce the risks of VAP. However, systematic investigation into the research trend and perspectives of applying EN for VAP patients is still lacking.

Therefore, this study aimed to explore the intellectual landscape of studies about the clinical application of EN in patients with VAP through scientometric analysis, and to identify thematic development trends, research frontiers and leading collaborations among researchers from various institutions. The study findings summarize the clinical benefits, potential risks and future research directions of the research field of EN for VAP treatment, provide evidence for future guideline development, and to potentially inspire future pre-clinical and clinical research for innovative EN products for treating VAP patients.

## Materials and Methods

### Dataset

Data from the Web of Science Core Collection Database was used in this study for two reasons. First, it provided references that met the format requirements for reference co-citation analysis as dictated by CiteSpace software. Second, the Web of Science Core Citation Database has a wide range of selective literature which might have a better citation rate. Therefore, this database is considered appropriate and sufficient to provide data that serves the purpose of this study.

Regarding data collection, the following retrieval strategy was developed: “TS = [(ventilator associated pneumonia or ventilator-associated pneumonia or ventilator acquired pneumonia or ventilator-acquired pneumonia) and (enteral nutrition)].” Language was set as “English”; literature category as “Article”; time from 1996/01/01 to 2018/8/23.

Using this search criteria, 124 pieces of literature were identified. The “Full Record and Cited References” of these records were also extracted as format of “Plain Text” into CiteSpace software. No duplicate records were identified using the native function of checking duplicate of the software. Therefore, the 124 literature papers were used as the final dataset.

### Software and Parameter Setting

CiteSpace software is an information visualization software developed by Professor Chaomei Chen using Java language. The software can visualize the structure, regularity and distribution of scientific knowledge (Chen, 2006). The main functions of CiteSpace software include co-citation analysis of literature, knowledge clustering and distribution of citation space, and co-occurrence analysis of knowledge units. Since its introduction, it has been widely adopted for scientometric analysis in various scientific fields around the world (Ping et al., 2017). In this study, the version of CiteSpace 5.3.R2 was used. The analysis parameters included:

• Modularity: The Modularity value evaluates the network modularity, ranging from 0 to 1. The larger the value, the better the cluster obtained by the network. When the Modularity value is greater than 0.3, the resulting network community structure is regarded as significant.
• Silhouette: The Silhouette value measures the homogeneity of the network, ranging from -1 to 1. The closer the value is to 1, the higher the homogeneity of the network. When the Silhouette value is greater than 0.5, the clustering result is considered reasonable. When the Silhouette value is greater than 0.7, the clustering result is believed to have high reliability.
• Time slicing: For the selection of time slices, we compared the clustering when the time slice was selected for 1 year or for 2 years. The time slice of 1 year was used because the Modularity value and the Silhouette value of the clustering effect was higher.
• Connection strength: For the connection strength, Cosine was used. For the threshold, the top 50 nodes in each time slice were selected.
• Pruning and merging: The pruning used pathfinder and the merged network.

### Data Analysis 1: Reference Co-citation Analysis

The co-citation relationship exists when two documents appear together in the bibliography of the third document. The mining process of the co-citation relationship of a document spatial data set is defined as reference co-citation analysis. Since the knowledge base consists of a collection of co-cited documents, the co-citation analysis of the literature was used to identify the knowledge base of a research field.

In this study, after setting the parameters, the “reference” function of CiteSpace software was used to visually analyze and cluster the data. The clustered Modularity and Silhouette values were found to be 0.841 and 0.6385, respectively, showing significant network community structure and reasonable reliability.

### Data Analysis 2: Key Words Co-occurrence Analysis

Key words co-occurrence analysis refers to word frequency analysis, which is a method of extracting the frequency and the high or low distribution of key words or subject words. This can help indicate the core content of the literature to report the development trends and research hotspots in this field. Co-word analysis is used to measure the frequency of a group of at least two words appearing in the same group of documents to measure their affinity. The key word co-occurrence analysis is provided by the authors in the dataset. This study used the key word co-occurrence analysis to show the evolution path, research hotspots and research frontiers. The research hotspot refers to a topic with time characteristics that is shared by scholars in a certain field. The research frontier is interpreted as the emerging theoretical trend and a new research theme.

The “keyword” function of CiteSpace software was used to perform visual analysis by clustering analysis. Through clustering of key words co-occurrence, the Modularity and Silhouette values were found to be 0.6995 and 0.6849, respectively, showing good effect of clustering.

### Data Analysis 3: Cooperation Network Analysis

If a paper has authors from different institutions, or countries, it suggests possible scientific partnership among these authors, institutions, and countries. Through the three-level research of national cooperation network, institutional cooperation network and researcher cooperation network, this study intended to present an overview of the research cooperation involved in the field of applying EN for VAP patients.

For this study, in the main interface of CiteSpace software, the nodes “country,” “institution,” “institution” + “author” were used, respectively, to visualize the three levels of scientific cooperation network. For the analysis of “institution” + “author,” in addition to the cooperation among the authors themselves, the relationship among the institutions and authors was also displayed.

## Results

### Number of Publications on EN Application for VAP Over Years

**FIGURE 1:**
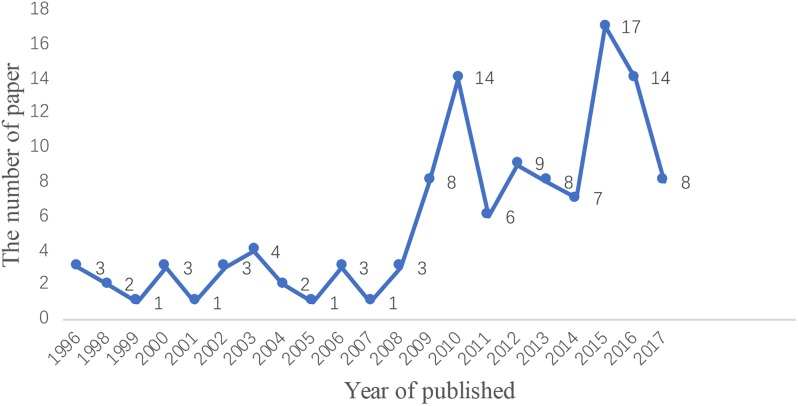
Number of publications on the application of EN in VAP patients over time. Curve represents the number of publications.

The average number of publications per year during the study period was less than 10, suggesting that the previous research surrounding EN application for VAP patients was limited. As shown in Figure 1, although there were only a few publications before 2009, an increase in the number of publications in recent years has been observed, implying a mounting academic interest in this research field.

### Reference Co-citation Analysis

**FIGURE 2:**
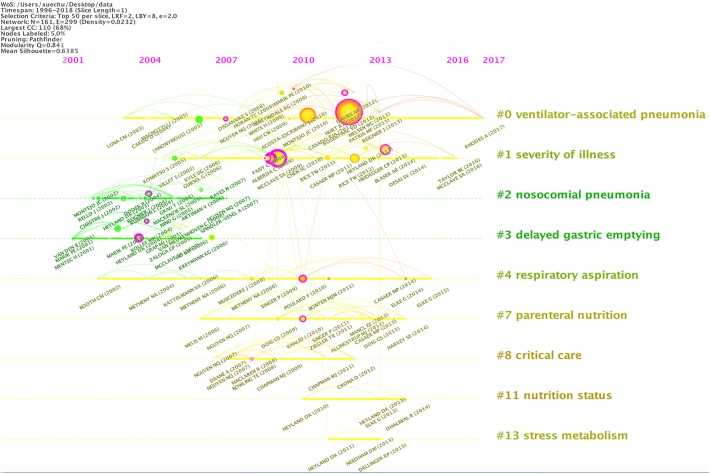
Clustering of reference co-citation of publications on the application of EN in VAP patients over time. The bigger the circle, the more research on the subject.

Figure 2 shows the clustering results of reference co-citation over time and Table 1 summarizes the nine main clusters of reference co-citation analysis. It is apparent that the majority of this research was carried out post-2001. The earlier research direction lay in cluster #2 “nosocomial pneumonia” and cluster #3 “delayed gastric emptying,” with most of the publications dated around 2004. This was followed by cluster #4 “respiratory aspiration” and cluster #8 “critical care,” and the related publications were mostly dated around 2008. Clustering #0 “ventilator-associated pneumonia” is the subject of this study, of which clustering time was from 2003 to 2017, with a larger number of publications around 2009. This was followed by cluster #1 “severity of illness” and cluster #7 “parenteral nutrition,” of which the publications dated mostly around 2010. Cluster #11 “nutrient status and clustering” and cluster #13 “stress metabolism” were the focus of the most recent publications dated around 2012. More detailed information about each cluster is presented as follows:

• **Cluster #2 “nosocomial pneumonia”**: The literature in this cluster describes the results and the impact of EN application for treating VAP patients, focusing on early EN intervention.
• **Cluster #3 “delayed gastric emptying”**: This cluster is mainly about the effect of various EN feeding pathways on the proportion of VAP patients. The overall recommended feeding method was via small intestine feeding. Conversely, nasal gastric tube feeding is associated with a higher risk of VAP. In addition, the clinical results of gastric feeding and post-pyloric feeding were not significantly different. Discussing the appropriate EN feeding pathways was the focus of research in this field from 2001 to 2005. It should be noted that this cluster has two articles published in 2005 that did not support the use of residual gastric volume as a marker of aspiration risks.
• **Cluster #4 “respiratory aspiration”**: It mainly refers to incorrect inhalation. Respiratory aspiration is one of the risk factors for VAP. The literature in cluster #4 focused on how to monitor, reduce or prevent aspiration in patients treated with EN and mechanical ventilation to reduce the prevalence of aspiration pneumonia. This research topic has been a long-standing research hotspot.
• **Cluster #8 “critical care”**: This cluster mainly discussed the problems faced by critically ill patients when they used EN, and focused on the situation of gastric emptying. The cluster has the highest consistency of literature.
• **Cluster #0 “ventilator-associated pneumonia”**: The study covers VAP therapy, benefits and risks of EN support for patients with mechanical ventilation, different EN feeding routes and duration for severe patients, residual gastric volume, effect of VAP rate on patients with ventilation, and the risk factors for the prevalence of VAP in pediatric ICU.
• **Cluster #1 “severity of illness”**: The primary concern is the use of EN in critically ill adults (including VAP patients), including the relationship between nutritional intake and clinical outcomes, appropriate nutrient intake, nutrient ratio, and timing of intake (early EN), combination of EN and parenteral nutrition, feeding intolerable, EN risk of obtaining VAP in mechanically ventilated patients. Critically ill patients were the primary focus in the field of EN applying for VAP.
• **Cluster #7 “parenteral nutrition”**: The literature in this cluster primarily concerns the parenteral nutrition support. In addition to nutritional support, it also included the comparison of parenteral nutrition and EN, parenteral nutrition as a supplement to EN, and clinical results of parenteral nutrition use. However, most of the literature papers in this cluster are not highly correlated with VAP.
• **Cluster #11 “nutrition status”**: This clustering literature covers improvements in nutritional support and related experiments.
• **Cluster #13 “stress metabolism”**: This clustering literature covers the study of nutrients (best calories) in nutritional support.

**Table 1:** Nine main clusters of reference co-citation analysis of publications on the application of EN in VAP patients over time.

Cluster ID	Size	Silhouette	Mean year	Label (LLR)
#2	17	0.916	2004	Nosocomial pneumonia
#3	13	0.883	2004	Delayed gastric emptying
#4	12	0.945	2008	Respiratory aspiration
#8	8	0.986	2008	Critical care
#0	23	0.756	2009	Ventilator-associated
				pneumonia
#1	19	0.892	2010	Severity of illness
#7	11	0.982	2010	Parenteral nutrition
#11	4	0.987	2012	Nutrition status
#13	3	0.979	2012	Stress metabolism

In general, the prime research interests around 2004 were mainly about the problems encountered when using EN in VAP patients and the feeding methods of EN. The research focus was subsequently shifted around 2008 to the gastric nutrition intolerance. Aspiration pneumonia has been a longer-term, active research area. Improvements in nutritional support and optimization of nutrients have become the major focus in recent studies.

### Highly Cited Articles

**FIGURE 3:**
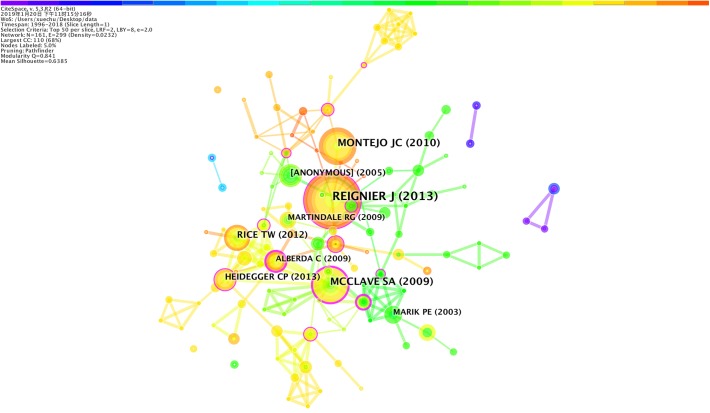
Clustering visualization of highly cited publications on the application of EN in VAP patients. The bigger the circle, the more citation of the publication.

The highly cited articles were clustered to identify the key knowledge base of the field of EN application for VAP patients. The visualization of clustering highly cited articles was presented in Figure 3.

The top 10 highest cited articles were summarized in Table 2. Among the top 10 articles cited, there were five clinical studies, four guidelines, and one systematic review. In terms of the topics of these papers, five studies focussed on critical care patients; three studies on residual gastric volume; one study on early nutritional care; and one study on EN. Overall, nutritional care for critically ill patients was the focus of all these studies. Highly cited literature was published mainly after 2009 in high-profile, international clinical medical journals such as *JAMA* and *Critical Care*.

**Table 2:** Top 10 highest cited publications on the application of EN in VAP patients.

Cluster ID	Citation counts	Authors	Summary	Year	Journal
#0	24	Reignier et al., 2013	To make it clear whether monitoring residual gastric volume affects the treatment of patients with mechanically ventilated pneumonia supported by enteral nutrition	2013	JAMA-J. Am. Med. Assoc., 309, 249
#0	16	Montejo et al., 2010	To compare the effects of increasing the limit for gastric residual volume (GRV) in the adequacy of enteral nutrition.	2010	Intens. Care Med., 36, 1386
#1	15	McClave et al., 2009	To provide guidance for nutritional support treatment for critically ill patients in adults.	2009	JPEN-Parenter. Enter., 33, 277
#1	11	Rice et al., 2012	To determine if initial lower-volume trophic enteral feeding would increase ventilator-free days and decrease gastrointestinal intolerances compared with initial full enteral feeding.	2012	JAMA-J Am. Med. Assoc., 307, 795
#0	10	American Thoracic Society and American Society of Infectious Diseases.	To provide guidance for the management of HAP, VAP and HCA to promote the use of appropriate doses of early antibiotics.	2005	Am. J. Resp. Crit. Care, 171, 388
#1	9	Heidegger et al., 2013	To optimize clinical outcome the researchers assessed whether delivery of 100% of the energy target from days 4 to 8 in the ICU with EN plus supplemental parenteral nutrition (SPN).	2013	LANCET, 381, 385
#3	8	Marik and Zaloga, 2003	To evaluate the impact of gastric versus post-pyloric feeding on the incidence of pneumonia, caloric intake, intensive care unit (ICU) length of stay (LOS), and mortality in critically ill and injured ICU patients.	2003	Crit. Care, 7, 0
#1	8	Alberda et al., 2009	To examine the relationship between the amount of energy and protein administered and clinical outcomes, and the extent to which pre-morbid nutritional status influenced this relationship.	2009	Intens. Care Med., 35, 1728
#0	8	Martindale et al., 2009	To examine the relationship between the amount of calories administered and mortality.	2009	Crit. Care Med., 37, 1757
#3	7	Kreymann et al., 2006	To provide evidence-based recommendations for patients with a complex course of illness during ICU hospitalization, with a particular focus on those patients with a severe inflammatory response, i.e., patient at least with one organ failure during ICU hospitalization.	2006	Clin. Nutr., 25, 210

### Articles With Citation Burst

Table 3 presents the articles with citation burst (a higher citation rate than a similar article published in the same period). The topics included the influence of body positioning on the prevalence of VAP, gastric colonization, VAP epidemiology, differences in different EN feeding pathways, and the effect of low calorie on disease outcomes. Excluding the guidelines and VAP epidemiological study, the papers were divided into three groups: the stomach colonization (1996–2003); the difference among various EN feeding pathways (2001–2010); and the effect of low calorie on disease outcomes (2007–2014).

**Table 3:** Top 16 articles with citation burst of publications on the application of EN in VAP patients.

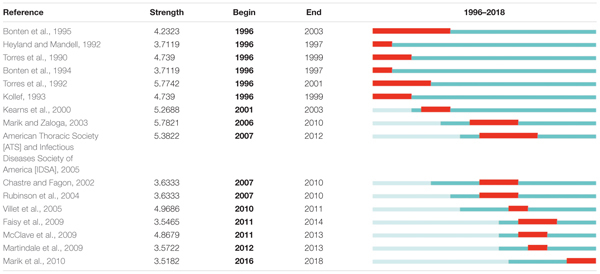

In particular, the most notable articles with citation burst were by: Torres et al. (1992) who studied the effect of body position on lung inhalation of stomach contents (aspiration); Kearns et al. (2000) who showed that the protein delivery rate of small intestine feeding was superior to that of gastric tube feeding; Marik and Zaloga (2003) who reported that there was no significant difference in clinical outcomes between pylorus and gastric tube feeding but gastric tube feeding was faster; and American Thoracic Society [ATS] and Infectious Diseases Society of America [IDSA] (2005) who introduced the adult management guidelines for hospital accessibility, ventilator-related and medical-related pneumonia. In addition, the work of Marik et al. (2010) focused on the benefits and risks of stress ulcer prevention and the regulation of EN, which received citation burst since in 2016, suggesting a potential leading research direction in this area.

### Key Words Co-occurrence as Indicators for Research Front

**FIGURE 4:**
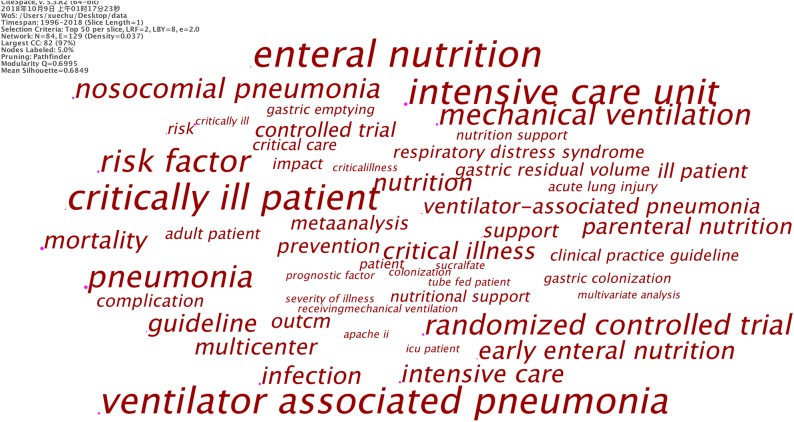
Key words with high frequency. The bigger the key words, the more frequently they appear in the literature.

Figure 4 shows the high co-occurrence key words when Threshold of CiteSpace software was set as 3. The larger the character, the higher the frequency of occurrence of the key word. It can be seen from Figure 4 that “ICU” and “critically ill patient” (or “critical illness”) were the main research targets in the field of clinical EN application for VAP patients. Risk factor, parenteral nutrition, early EN, infection, mortality, residual gastric volume, gastric emptying, and gastric cavity colonization bacteria were the main research focuses in this field. Randomized controlled trial was the main research method in this field, in addition to meta-analysis.

### Key Words With Burst Impact

Table 4 shows the top 12 key words with burst impact. “Bacterial colonization,” “gastric colonization,” and “pneumonia” were the three key words that had the earliest burst impact. “Pulmonary aspiration” (mainly referring to inhalation of stomach contents which causes aspiration pneumonia) and “body positioning” (referring to the relevant research that raises the bedside 45 degrees to help reduce VAP) had burst impact during 1999–2001. The most bursting key words were “risk factor,” “gastroesophageal reflux,” and “parenteral nutrition,” which are also the latest bursting key words.

**Table 4:** Top 12 key words with burst impact according to the publications on the application of EN in VAP patients over time.

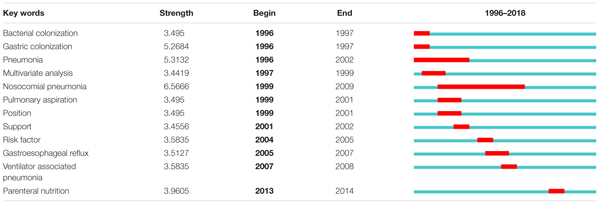

### Cooperation Network Among Countries

As shown in Table 5, the United States had the highest publication number, followed by Canada and France. These countries had continuous research in the field of applying EN for VAP patients. Research originating in China, Turkey and Germany in this field started to emerge since 2014.

**Table 5:** Top 10 countries in the field of EN for VAP regarding publication number.

Publication number	Country	Beginning year
35	United States	2004
12	France	2009
10	Canada	2012
6	China	2015
4	Spain	2010
3	Turkey	2016
2	Germany	2014
2	Taiwan	2012
2	Japan	2012
2	Netherlands	1996

**FIGURE 5:**
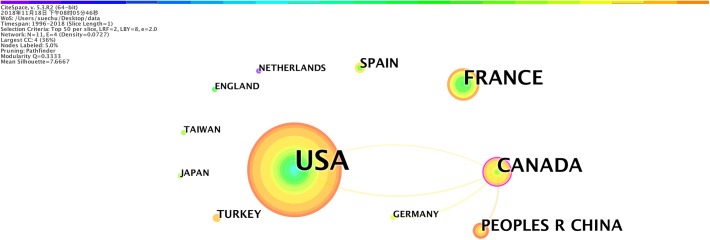
The major countries of origin and collaborative activities regarding the research about the application of EN in VAP patients. The bigger the circle, the more original research.

However, as shown in Figure 5, there was limited scientific cooperation among these above-mentioned countries in the field. While the United States carried out most of the research alone, Canada was the only country that played a central role to facilitate collaboration with United States, China, and Germany.

### Cooperation Network Among Institutions

Regarding the publication number per institution, as shown in Table 6, Queens University from Canada had the most publications. Interestingly, being the country with the largest number of publications, the United States had only one institution in the top 10 list, which might indicate that the research institutions in this field were more dispersed in the country.

**Table 6:** Top 10 institutions regarding publication number.

Publication		Country	Beginning
number	Institution	(area)	year
5	Queen’s University	Canada	2012
4	Kingston General Hospital	Canada	2015
2	University Hospital Maastricht	Netherlands	1996
2	Eemland Hospital	Netherlands	1996
2	Taipei Veterans General Hospital	Taiwan	2012
2	Dist. Hosp. Ctr.	France	2010
2	University of California, Los Angeles	United States	2009
2	Hospital General University of Alicante	Spain	2010
2	National Cheng Kung University	Taiwan	2012
2	Hotel-Dieu de France University Hospital	France	2010

**FIGURE 6:**
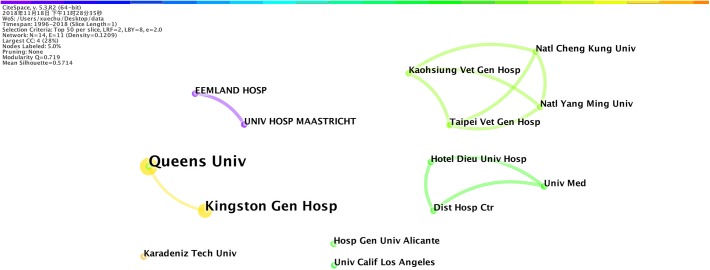
Cooperation network among institutions on the research about the application of EN in VAP patients. Lines represent cooperation network among institutions.

As shown in Figure 6, scientific cooperation among institutions was greatly influenced by the geographical location, and cooperation basically occurred among institutions in the same country (area). The types of institutions involved in research in this field were mainly universities and hospitals. The close cooperation between universities and hospitals was the main cooperation form in this research field.

### Cooperation Network Among Researchers

As shown in Table 7, Heyland DK was the leading researcher in the field of studying EN application for VAP, who had published eight papers in this field since 2012. Meanwhile, the top 10 authors were mostly not consistent with the authors of the highest cited articles. Among these top 10 researchers, only one of Reingier J’s articles was one of the top 10 highest cited articles.

**Table 7:** Top 10 researchers regarding publication number.

Publication number	Researcher	Beginning year
8	Heyland DK	2012
2	Bonten MJM	1996
2	Saylan S	2016
2	Vandergeest S	1996
2	Reignier J	2010
2	Chang SJ	2012
2	Kang SP	2012
2	Stobberingh EE	1996
2	Liu MY	2012
2	Bengmark S	2009

**FIGURE 7:**
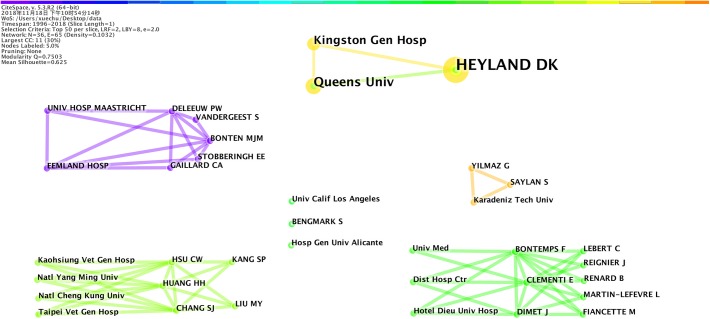
Cooperation network among researchers on the research about the application of EN in VAP patients. Lines represent cooperation network among researchers.

As shown in Figure 7, the cooperation network at researcher level and institution level in this field was mostly consistent. The researchers’ cooperation network was highly correlated with the cooperation network of the corresponding institutions as identified in Figure 6.

## Discussion

Our study used a scientometric research method to map the structure and the evolution trend of the research about the application of EN in VAP patients. By combining reference co-citation analysis, key words co-occurrence analysis and cooperation network analysis, this study found that this research field has evolved from studying feeding pathways and risk factors, to exploring the optimization of EN use in VAP patients. These three aspects raised some issues that are discussed further as follows.

Firstly, at the early development phase of this field, researchers generally agreed that clinical EN application had benefits for VAP patients based on findings from clinical experiments. However, researchers were concerned about the impact of different feeding pathways of EN. After a series of further clinical research, a consensus was reached that small intestine feeding should be recommended (Heyland et al., 2004). This consensus was reflected in some guidelines of EN application in VAP.

Secondly, researchers began to pay attention to the possible risks associated with EN interventions in VAP patients, which were often related to the patient’s gastric emptying. Gastric emptying is an important indicator for EN intervention. Delaying gastric emptying can lead to nutritional intolerance and gastric reflux that can increase the risk of aspiration. The research on delayed gastric emptying focused on influencing factors such as cholecystokinin (CCK) and peptide YY (PYY), admission diagnosis, and blood glucose concentrations. Literature identified that red mold and metoclopramide could promote tolerance to EN in the stomach, and erythromycin might enhance gastric motility more effectively than metoclopramide (MacLaren et al., 2008). Based on these findings, it was recommended that health professionals should monitor possible delaying gastric emptying during clinical application of EN for VAP patients.

In addition, aspiration is one of the main causes of VAP and also a potential risk of EN application. Studies focusing on how to avoid aspiration have been ongoing. At present, the recommended method is to use a standard polymeric enteral formulation. This formulation should begin within 24 to 48 h of admission to ICU. The patient is treated in semi-recumbent positioning and should not use ammonia-containing acidic enteral products. Other recommendations include: (1) Start with the target rate by using a higher threshold of gastric residuals; (2) Use feeding regimens such as exercise agents and small bowel feeding to optimize EN delivery; and (3) Raise bedside measures to minimize the risk of EN. As the cause of aspiration is uncertain, how to completely prevent and control the occurrence of aspiration remains challenging. The cause of aspiration and the measures to reduce the risk of aspiration has now become the prime research focus in this field.

Thirdly, the optimization of EN interventions in VAP patients, which appeared around 2011, is a relatively new research topic. This topic focuses on identifying the most suitable EN intervention for VAP patients through clinical experiments. EN optimization includes optimization of EN feeding methods and formulation ingredients. For example, according to prospective studies on patients with mechanical ventilation, patient survival was closely related to calorie satisfaction, and a suitable intestinal route feeding regimen to provide an enhanced protein-energy supply was clinically significant (Heyland et al., 2011; Heyland, 2013). Another Canadian study, which attempted to shift from an hourly target rate to a 24-h target rate, suggested the use of trophic feeding to start exercise agents and protein supplements on the first day of EN intervention in order to increase the residual gastric volume threshold (Heyland et al., 2010). At present, experiments in this area are scarce, and the number of patients was also insufficient. This topic will remain to be the key research direction for EN intervention in VAP patients in the near future.

### Research Implications

The scientometric analysis employed in this study has some important implications to clinical practice and academic research. First, clinical practitioners need to pay more attention to the risks of EN usage for VAP. Because EN is dependent on intestinal absorption, several risk factors, such as aspiration, could generate a negative impact on VAP patients. Therefore, the risks factors, especially delayed gastric emptying, should be closely monitored by clinical practitioners when using EN. Second, our study found that the guidelines have an important place in the field. However, the guidelines for the application of EN in VAP patients that is currently available was published in Canada back in 2003. Given the new research results in the past 10 years, it is necessary to revise and update the guideline accordingly. Third, co-authorship research shows that there is little cooperation among the countries, and cooperation is severely restricted by geographical factors. International cooperation in this field should be promoted further. Direct cooperation among hospital, university and industry should be encouraged to accelerate the development and clinical test of innovative EN products.

### Strength and Limitations

To our knowledge, this is the first study to systematically analyze the research surrounding EN application in VAP patients using the scientometric method. However, this study also has some limitations which can be addressed in future studies. First, to meet the reference format requirement of CiteSpace software, we only retrieved literature from Web of Science Core Collection Database in this study. Future study can extend to include other literature databases, such as PubMed database through co-occurrence key words detection. Second, as many problems of EN interventions for VAP have not been reported academically, opinions (or experience) from clinical experts reported in the current literature may be incomplete, warranting further research to enrich the knowledge of this field.

## Conclusion

The advantages of applying EN for VAP has been recognized by current literature. However, this field is at its early developing stage. Optimization of EN use for VAP patients is the key research direction now and in the near future. Strategic cooperation among hospital physicians, university researchers and industry developers should be encouraged to establish consistent clinical guidelines and develop innovative EN products for VAP patients.

## Data Availability

All datasets generated for this study are included in the manuscript and/or the supplementary files.

## Author Contributions

SC, RB, YL, CU, and HH conceived and designed the study. RB and YL collected the data and performed software analysis. SC, RB, HS, and HH drafted the first vision. YL and CU revised the manuscript critically. All authors reviewed and approved the final manuscript.

## Conflict of Interest Statement

The authors declare that the research was conducted in the absence of any commercial or financial relationships that could be construed as a potential conflict of interest.
